# A nomogram to predict severe COVID-19 patients with increased pulmonary lesions in early days

**DOI:** 10.3389/fmed.2024.1343661

**Published:** 2024-04-26

**Authors:** Lina Chen, Min Li, Zhenghong Wu, Sibin Liu, Yuanyi Huang

**Affiliations:** ^1^Department of Radiology, Jingzhou Hospital Affiliated to Yangtze University, Jingzhou, Hubei Province, China; ^2^Department of Radiology, Jingzhou Hospital of Traditional Chinese Medicine, Jingzhou, Hubei Province, China

**Keywords:** COVID-19, tomography, X-ray computed, pneumonia, viral, artificial intelligence, prognosis, nomograms

## Abstract

**Objectives:**

This study aimed to predict severe coronavirus disease 2019 (COVID-19) progression in patients with increased pneumonia lesions in the early days. A simplified nomogram was developed utilizing artificial intelligence (AI)-based quantified computed tomography (CT).

**Methods:**

From 17 December 2019 to 20 February 2020, a total of 246 patients were confirmed COVID-19 infected in Jingzhou Central Hospital, Hubei Province, China. Of these patients, 93 were mildly ill and had follow-up examinations in 7 days, and 61 of them had enlarged lesions on CT scans. We collected the neutrophil-to-lymphocyte ratio (NLR) and three quantitative CT features from two examinations within 7 days. The three quantitative CT features of pneumonia lesions, including ground-glass opacity volume (GV), semi-consolidation volume (SV), and consolidation volume (CV), were automatically calculated using AI. Additionally, the variation volumes of the lesions were also computed. Finally, a nomogram was developed using a multivariable logistic regression model. To simplify the model, we classified all the lesion volumes based on quartiles and curve fitting results.

**Results:**

Among the 93 patients, 61 patients showed enlarged lesions on CT within 7 days, of whom 19 (31.1%) developed any severe illness. The multivariable logistic regression model included age, NLR on the second time, an increase in lesion volume, and changes in SV and CV in 7 days. The personalized prediction nomogram demonstrated strong discrimination in the sample, with an area under curve (AUC) and the receiver operating characteristic curve (ROC) of 0.961 and a 95% confidence interval (CI) of 0.917–1.000. Decision curve analysis illustrated that a nomogram based on quantitative AI was clinically useful.

**Conclusion:**

The integration of CT quantitative changes, NLR, and age in this model exhibits promising performance in predicting the progression to severe illness in COVID-19 patients with early-stage pneumonia lesions. This comprehensive approach holds the potential to assist clinical decision-making.

## 1 Introduction

The infection of coronavirus disease 2019 (COVID-19), caused by severe acute respiratory syndrome coronavirus 2 (SARS-CoV-2), rapidly became a global pandemic. The virus spread extensively in China and various other regions (1). While current vaccines mitigate the impact of COVID-19, humanity should remain prepared for potential future pandemic threats that could endanger the global population (2). Like other rapidly expanded pneumonia, in most COVID-19 patients, pneumonia lesions were observed to have a rapid progression in 1 week (3). The critically ill patients had a high fatality ratio (4). Thus, it is very important to identify high-risk patients in the early stages to adopt different prevention and treatment efforts.

Pneumonia lesions were observed earlier than clinical symptoms in most COVID-19 patients (4). This indicated that computed tomography (CT) can be used in early diagnosis and monitoring COVID-19 (5). Compared to CT feature score and radiomics, artificial intelligence (AI)-derived quantified lung CT scans offer the advantages of objectivity, ease of accessibility, and time efficiency (6–8). Currently, many clinical features, including clinical factors and laboratory examinations, have been identified as prognostic risk factors (9). Some published studies indicated that CT features or CT features combined with clinical features outperformed the traditional clinical features alone in prognosis prediction (10–12). However, most of these studies focused on CT imaging obtained at the time of hospital admission, while neglecting the pre-admission data for COVID-19. Few studies have noticed the potential prognostic value of changes occurring in the short term. However, these studies have not addressed excluding negative changes, like the volume decrease of pneumonia lesions in their analysis (13, 14).

Therefore, our study proposes a simple nomogram that has the potential to predict severe COVID-19 patients who have increased pulmonary lesions in the early days of infection. The nomogram is designed to use quantitative lung CT data to evaluate the severity of lung damage in COVID-19 patients.

## 2 Materials and methods

### 2.1 Patients

**Figure 1:**
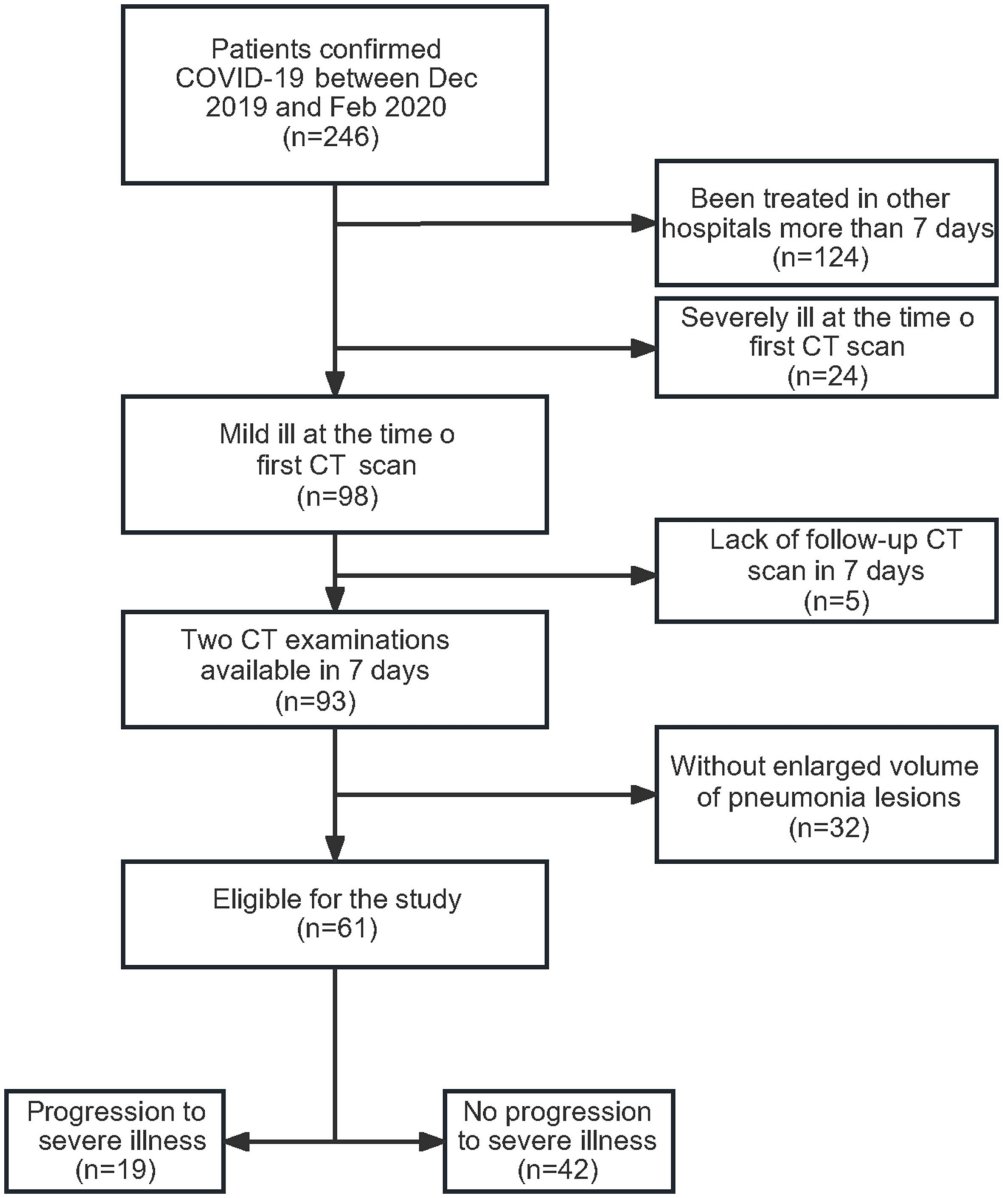
Flow diagram of the study population.

A total of 246 patients were diagnosed with COVID-19 infection by SARS-CoV-2 nucleic acid test in Jingzhou Central Hospital between 17 December 2019 and 20 February 2020. The patients who had been treated in another hospital for more than 7 days (*n* = 124) were diagnosed severely ill at the time of the first chest CT scan (*n* = 24), and missed follow-up chest CT scans in early 7 days (*n* = 5) were excluded in this study (Figure 1). Among the remaining 93 patients, 61 exhibited enlarged pneumonia lesions, with an enlargement range exceeding 10 cm^3^. Then, all 61 patients were followed up for 30 days. At last, 42 showed no progression to severe illness and 19 exhibited progression to severe illness.

According to the guidelines of Chinese national diagnosis and treatment protocols for COVID-19 (15) and the guidelines of the American Thoracic Society (16), patients were defined as having severe illness if they reached any of the following endpoints: (a) multi-lobar infiltrates and respiratory failure (arterial oxygen pressure (PaO2) < 60 mmHg, accompanied or unaccompanied with PaCO2 > 50 mmHg); (b) mechanical ventilation, intensive medical care or extracorporeal membrane oxygenation (ECMO) treatment (c); organ damage; and (d) in-hospital mortality. In this study, organ damage include ARDS (according to the Berlin definition), acute cardiac injury (heart function graduation ≥ IV levels by New York Heart Association heart function rating), acute kidney injury (AKI, according to the Kidney Disease Improving Global Outcomes clinical practice guidelines), and liver dysfunction alanine (aminotransferase >5 times upper limit of normal) (7, 13).

### 2.2 Clinical data and CT examination collection

We collected the demographic, clinical, laboratory, and outcome data from electronic medical records in the hospital information system (HIS). The baseline characteristics, including sex, age, and comorbidities, were collected. In this study, the comorbidities include diabetes, hypertension, cardiovascular disease, cerebrovascular disease, chronic obstructive pulmonary disease (COPD), chronic hepatitis B infection, and malignant tumors. The examinations of initial and follow-up in 7 days include neutrophil-to-lymphocyte ratio (NLR) and C-reactive protein (CRP). Meanwhile, the changes between the two examinations were depicted.

Chest CT imaging, including initial and follow-up in 7 days, was acquired from the hospital Picture Archiving and Communication System (PACS). If examinations were collected from outpatient and inpatient departments, they should be matched by name, age, exam time, exam number, and ID card number (if applicable) to verify they belong to the same individual.

All CT examinations were performed using a 16-slice scanner (Siemens SOMATOM Emotion, Germany) without contrast injection when the patients held their breath. The position was supine with the head advanced. Imaging parameters were set as follows: 130 kV; automatic tube current; slice width, 5 mm; beam collimation, 1.2 mm; tube rotation time, 0.6 s; reconstructed slice thickness, 1.5 mm; and matrix, 512 × 512.

### 2.3 CT image review and AI-based quantization

**Figure 2:**
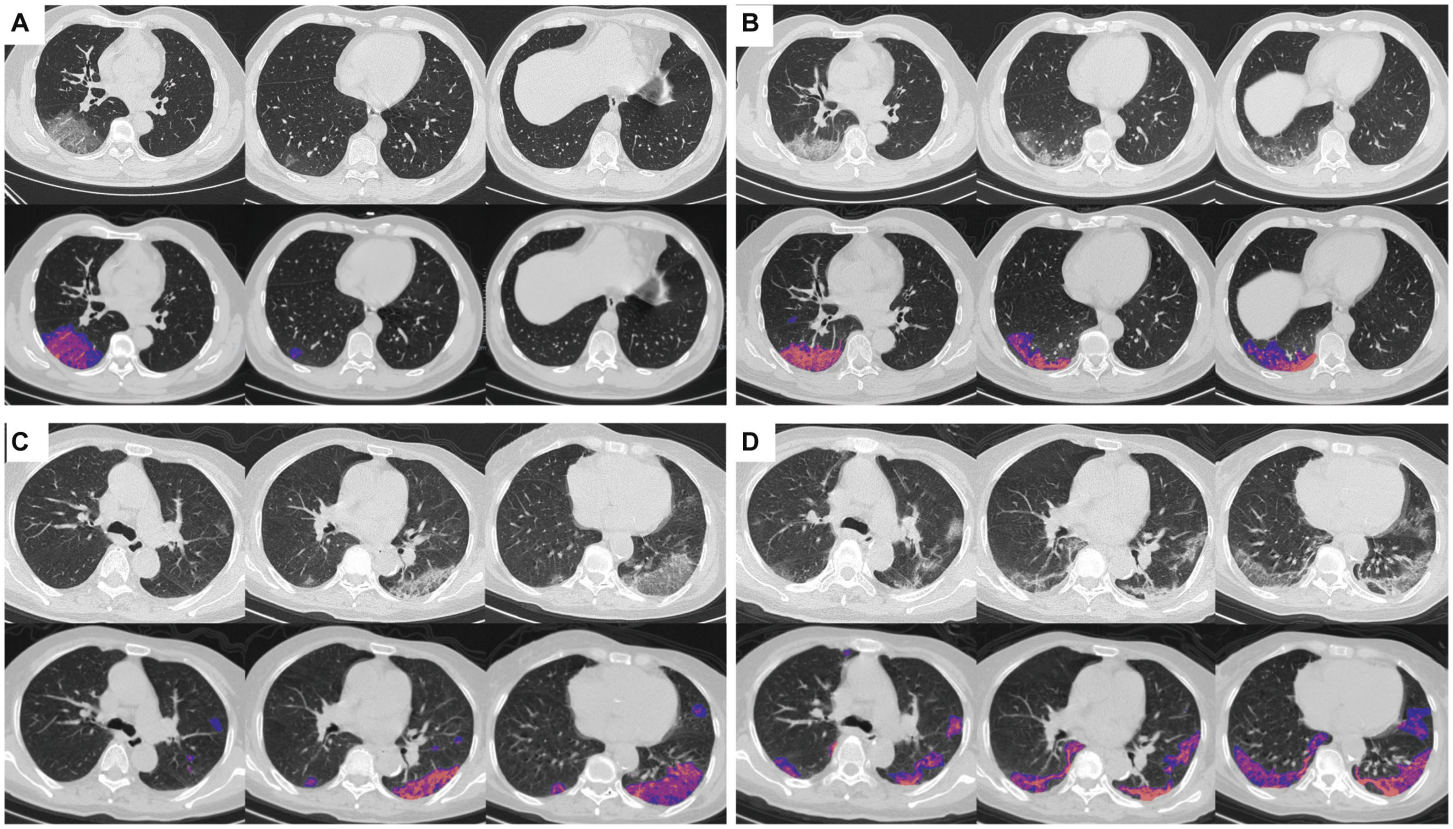
COVID-19 pneumonia lesions detected by the AI system and visualized as pseudo colors. First rows: initial CT images; second rows: AI auto-segmentation displayed with blue pink, and red pseudo colors representing ground-glass opacity (GGO), semi-consolidation and consolidation, respectively. **(A,B)** First CT imaging **(A)** and fellow-up after 3 days **(B)** of a 56-year-old male patient, who did not meet the endpoint during the follow-up. **(C,D)** First CT imaging **(C)** and fellow-up after 4 days **(D)** of a 80-year-old female patient, who reached the endpoint of progression to severe illness and died after 13 days.

Computed tomography images were automatically analyzed using an AI system (YT-CT-Lung, YITU Healthcare Technology Co., Ltd., China). This AI system utilized a fully convolutional network along with adaptive thresholding and morphological operations for the segmentation of lungs and pneumonia lesions (17, 18). Two experienced radiologists, each with over 10 years of experience, independently reviewed the CT images to ensure concordance with the AI-based segmentations corresponding to the lesion range in the CT scan. Illustrative examples of the automatically segmented pulmonary tissue and pneumonia lesions from COVID-19 are shown in Figure 2.

Besides the total volume of pneumonia lesions, other three quantitative features were computed by thresholding on CT values. The CT value range of −750 HU to −500 HU, −500 HU to −200HU, and −200HU to 60HU was defined as ground-glass opacity volume (GV), semi-consolidation volume (SV), and consolidation volume (CV), respectively. In this study, semi-consolidation refers to an intermediate, homogeneous area where there is a noticeable increase in density (19).

### 2.4 Statistical analysis

Patients were divided into two groups according to the results of follow-up, the severe group and the non-severe group. For only one NLR result that was not collected in this study, we used the median value to replace it. During the initial examination, five (8.2%) CRP results were missed, and three were missed during follow-up (4.9%). To address this issue of missing data, we conducted a chained equation approach method with five replications for multiple imputations using the R MI procedure.

We presented continuous variables as the median and interquartile range (IQR), while categorical variables as numbers and percentages. The Wilcoxon rank-sum test and chi-square tests were applied for relevant comparisons between severe and non-severe groups.

Binary logistic regression was used to explore the association between the predictive features and the development of severe illness. We performed a selection process by excluding all variables with *p*-values of ≥0.05 in the univariate regression analysis. Subsequently, we analyzed the remaining variables using multivariable regression. To simplify the model, we classified all the continuous variables based on quartiles and curve fitting results. Finally, we constructed a mixed nomogram model based on regression coefficients. The calibration of this model was evaluated through bootstrapping with 200 resamples to produce calibration curves and the Hosmer–Lemeshow goodness-of-fit test. The discrimination of the nomogram model was assessed by the receiver operating characteristic curve (ROC) and the area under the curve (AUC). The AUC in the prediction models was compared using the Delong non-parametric approach. The clinical benefits of the model were determined using decision curve analysis (DCA).

Two-tailed tests were conducted, considering *p* values of <0.05 as statistically significant. Statistical analyses were carried out using R version 3.3.2 (The R Foundation, https://www.r-project.org/) and the Free Statistics software package version 1.7.

## 3 Results

### 3.1 Clinical and CT quantitative characteristics

Of the total 93 COVID-19 patients, 32 presented reduced pneumonia lesions or increasing volume of less than 10 cm^3^, and 61 observed increased lesions in follow-up lung CT. There were no statistical differences in age, gender, or comorbidity between them. However, the progression to severe illness was statistically different, with a *p* value of 0.006 (Supplementary Table 1).

We classified 61 patients with lung lesions greater than 10 cm^3^ into two groups based on disease severity of follow-up: non-severe (*n* = 42) and severe (*n* = 19). Severe patients (54.0 years, IQR 42.0–60.0) were older compared to non-severe patients (38.5 years, IQR 32.2–49.8, *p* = 0.014). Although there was a higher proportion of males in the severe group compared to the non-severe group, this difference did not reach statistical significance (55.7 vs. 44.3%, *p* = 0.18). The presence of comorbidities was significantly associated with a worse outcome (*p* = 0.04). There was no significant difference in the duration between initial examinations and follow-up between the two groups.

In initial examinations, there were no significant differences for all clinic and CT features between severe and non-severe patients. However, in follow-up, NLR (*p* = 0.032) and CPR (*p* = 0.027) were significantly higher in the severe patients than in the non-severe patients. About CT features, except GV, the other three (lesion volume, CV, and SV) were significantly higher in the severe group than in the non-severe group (*p* < 0.05). Regarding the changes in 7 days, the range of increased lesion volume was from 10.71 to 958.69 cm^3^. All CT features, except GV, exhibited a more pronounced increase in the severe group, whereas the clinical biomarkers (NLR and CPR) did not exhibit any significant changes (Table 1).

**Table 1:** Clinical and CT quantitative characteristics of COVID-19 patients with enlarged lung lesions.

		Clinical progression		
Characteristic	Total (*n* = 61)	Non-severe (*n* = 42)	Severe (*n* = 19)	*p*
Age, year, (IQR)	44.0 (34.0, 55.0)	38.5 (32.2, 49.8)	54.0 (42.0, 60.0)	0.014
Gender, *n* (%)				0.18
Male	34 (55.7)	21 (50)	13 (68.4)	
Female	27 (44.3)	21 (50)	6 (31.6)	
Comorbidity, *n* (%)				0.04
No	43 (70.5)	33 (78.6)	10 (52.6)	
Yes	18 (29.5)	9 (21.4)	9 (47.4)	
Initial examination (IQR)				
RP^a^	12.3 (6.5, 29.1)	10.1 (4.7, 19.4)	16.8 (11.2, 46.2)	0.038
NLR^b^	2.7 (1.7, 4.1)	2.6 (1.7, 3.5)	3.7 (1.9, 5.2)	0.119
Lesion volume(cm^3^)	42.6 (15.2, 138.9)	42.4 (16.4, 98.1)	53.4 (10.3, 224.9)	0.907
GV	14.0 (4.8, 52.8)	13.9 (5.4, 32.3)	23.0 (3.9, 76.2)	0.895
SV	149 (2.5, 49.4)	15.0 (3.3, 34.7)	5.5 (1.5, 97.4)	1
CV	3.9 (0.6, 16.7)	3.9 (0.6, 8.9)	3.6 (0.5, 33.1)	0.602
Follow-up (IQR)				
CRP^c^	10.4 (3.2, 26.4)	8.7 (2.6, 21.4)	25.0 (8.1, 44.2)	0.027
NLR	5.9 (2.1, 16.7)	5.3 (2.0, 10.3)	16.7 (3.5, 21.6)	0.032
Lesion volume(cm^3^)	155.0 (70.5, 338.2)	142.2 (70.4, 243.9)	215.5 (88.0, 816.1)	0.233
GV	50.4 (26.7, 110.7)	49.7 (26.9, 84.4)	91.9 (26.3, 202.4)	0.3
SV	50.0 (17.4, 134.6)	47.6 (17.2, 80.5)	64.1 (28.9, 305.6)	0.145
CV	24.4 (5.9, 62.4)	19.5 (6.6, 44.4)	61.5 (5.0, 140.0)	0.141
Changes in 7 days (IQR)				
CRP	−2.5 (−12.1, 7.7)	−1.5 (−10.0, 4.0)	−6.5 (−20.7, 19.1)	0.697
NLR	2.1 (−0.4, 12.2)	1.5 (−0.4, 5.4)	10.7 (−0.6, 17.7)	0.237
Lesion volume(cm^3^)	92.4 (47.9, 210.2)	75.9 (46.1, 157.5)	210.2 (49.9, 359.9)	0.067
GV	36.4 (15.1, 54.4)	34.8 (14.3, 49.9)	38.0 (17.7, 89.3)	0.279
SV	25.4 (10.8, 63.2)	22.1 (9.5, 54.7)	59.4 (18.0, 157.6)	0.024
CV	12.2 (2.0, 41.9)	10.3 (1.4, 33.3)	39.6 (3.9, 74.0)	0.046
Interval, day, (IQR)	3.0 (3.0, 4.0)	3.5 (3.0, 4.0)	3.0 (3.0, 4.5)	0.954

^a^Five missing replaced using multiple imputations; ^b^One missing replaced by median value; ^c^Three missing replaced using multiple imputations. CRP, C-reactive protein; NLR, Neutrophil-to-lymphocyte ratio; GV, Ground-glass opacity volume; SV, semi-consolidation volume; CV, consolidation volume.

### 3.2 Relationships between clinical and CT quantitative characteristics and severe illness

The results of the univariate logistic regression and multivariable logistic regression are presented in Table 2. In follow-up, NLR, SV, and CV were significantly associated with progression to the severe illness of COVID-19 patients with increased pneumonia lesions in the early days (*p* < 0.05). However, calculating the change in 7 days, only CT characteristics (lesion volume, SV, and GV) exhibited a significant association with severe illness when assessing changes over 7 days. Notably, there were minimal disparities in the results between the univariate and multivariable logistic regression analyses.

**Table 2:** Logistic regression analysis of COVID-19 patients with enlarged lung lesions.

	Univariate regression		Multivariable regression^a^	
Characteristic	OR (95% CI)	*p*	OR (95% CI)	*p*
Age, year, (IQR)	1.07 (1.02 ~ 1.12)	0.01	1.07 (1.02 ~ 1.12)	0.009
Gender, *n* (%)				
Male	NA	NA	NA	NA
Female	0.46 (0.15 ~ 1.44)	0.184	NA	NA
No	NA	NA	NA	NA
Yes	3.3 (1.03 ~ 10.57)	0.044	3.06 (0.94 ~ 9.96)	0.063
Follow-up (IQR)				
CRP^c^	1.02 (1 ~ 1.04)	0.093	NA	NA
NLR	1.09 (1.02 ~ 1.16)	0.011	1.09 (1.02 ~ 1.16)	0.013
Lesion volume (cm^3^)	1 (1 ~ 1)	0.058	NA	NA
GV	1 (1 ~ 1.01)	0.226	NA	NA
SV	1.01 (1 ~ 1.01)	0.012	1.01 (1 ~ 1.01)	0.022
CV	1.01 (1 ~ 1.02)	0.032	1.01 (1 ~ 1.02)	0.048
Changes in 7 days (IQR)				
CRP	1.01 (0.99 ~ 1.03)	0.553	NA	NA
NLR	1.06 (0.99 ~ 1.13)	0.074	NA	NA
Lesion volume (cm^3^)	1 (1 ~ 1.01)	0.022	1 (1 ~ 1.01)	0.032
GV	1.11 (1.02 ~ 1.2)	0.02	NA	NA
SV	1 (1 ~ 1.01)	0.122	1.01 (1 ~ 1.02)	0.019
CV	1.01 (1 ~ 1.02)	0.014	1.01 (1 ~ 1.03)	0.048
Interval, day, (IQR)	1.01 (0.57 ~ 1.82)	0.961	NA	NA

^a^Adjusted of gender. CRP, C-reactive protein; NLR, Neutrophil-to-lymphocyte ratio; GV, Ground-glass opacity volume; SV, Semi-consolidation volume; and CV, Consolidation volume.

### 3.3 Development and performance of prediction model

**Figure 3:**
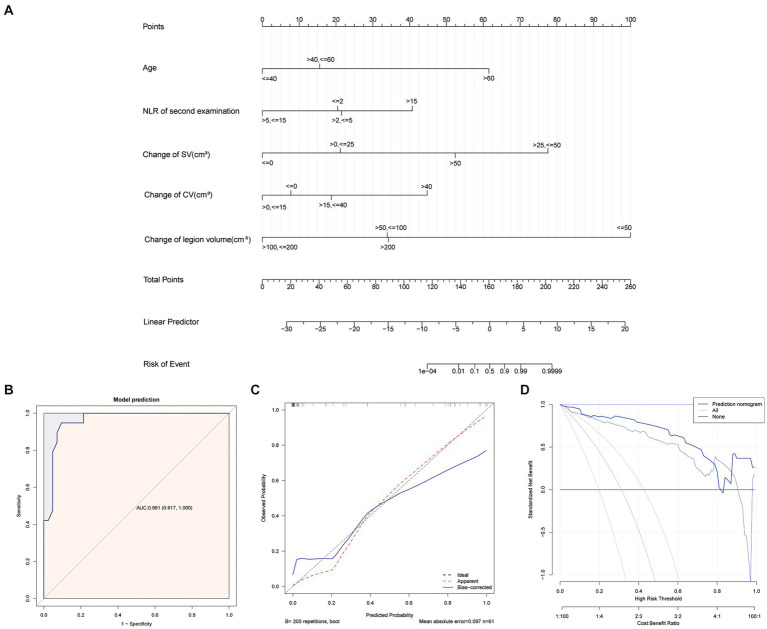
Development and performance of nomogram. A nomogram **(A)** for the prediction of developing severe illness of COVID-19 patients. ROC curves **(B)** of nomogram. Calibration curves of the nomogram **(C)**, which depict the calibration of the nomogram in terms of the agreement between the predicted risk of severe illness and observed outcomes. The 45°dotted blue line represents a perfect prediction, the dotted red lines represent the predictive performance of the nomogram, and the blue lines represent the bootstrap validation. The closer the dotted red and blue line fit is to the ideal line, the better the predictive accuracy of the nomogram is. Decision curve analysis (DCA) for the nomogram **(D)**, the *y*-axis represents the net benefit. The blue line represents the AI mixed nomogram. The gray line represents the hypothesis that all patients had developing severe illness. The black line represents the hypothesis that all patients had no progression to severe illness. The *x*-axis represents the threshold probability.

In our study, the initial examination NLR and changes over 7 days were not significantly associated with progression to severe illness in COVID-19 patients with escalating pneumonia lesions during the early days. However, NLR in the follow-up examination exhibited a significant relationship. According to the clinical experiments, we added it to the mixed model. Finally, age, NLR in the second blood test, and changes in quantitative CT features (lesion volume, SV, and CV) between the two examinations were selected based on the multivariable logistic regression analysis. Combining quartiles and curve fitting results, a mixed model was developed and presented as a nomogram (Figure 3).

The model showed the highest discrimination between severe COVID-19 patients and no-severe patients in those who had increased lung lesions in the early days, with an AUC of 96.05% (91.66–100%). The sensitivity, specificity, and accuracy of the model were 94.74%, 90.48%, and 0.918, respectively.

The calibration curve of the nomogram showed strong agreement between the severe illness predicted and the mixed model (Figure 3C). The Hosmer–Lemeshow test resulted in a *p* value of 0.666, indicating a good fit for the nomogram. The bootstrap validation technique yielded a *p* value of 0.854, confirming the model’s acceptable performance.

Figure 3D illustrates the DCA, demonstrating that the use of a nomogram in predicting severe illness provided a higher net benefit compared to both the “treat all” and “treat none” strategies across threshold probabilities ranging from 1 to 81% in the cohort. This highlights the clinical utility of the nomogram.

There was a significant difference in predictive efficacy between the mixed model with categorical variables and with continuous variables (*p* = 0.009) (Supplementary Figure 1).

## 4 Discussion

In our study, we developed a quantitative CT feature-based model that incorporates age, and NLR from follow-up examinations within a 7-day period. This model could predict the risk of progressing to severe illness in COVID-19 patients who had an increase in pneumonia volume exceeding 10 cm^3^ within a 7-day timeframe. The results showed that quantitative CT features of two examinations, as well as their changes, could predict the risk of COVID-19 patients progressing to severe illness. The nomogram demonstrated favorable discrimination (AUC, 0.961) and good calibration. DCA indicated the clinical usefulness of the mixed model.

We investigated to evaluate the potential features for predicting severe illness. This assessment uses three data points: the examination data from both outpatient and inpatient departments when the patients arrive at the hospital for the first time, a second data point obtained within a maximum interval of 7 days from the first examinations, and the changes observed between two points. Our study has extended the observation period of the disease in comparison to previous studies that only utilized admission data (13).

Given their previously reported prognostic potential (20–22) and the feasibility of routine blood analysis, we selected NLR and CPR as representative laboratory biomarkers for comparison in this study. We observed improved performance of NLR and quantitative CT features in the second examination when the interval between the two inspections was less than 7 days. This observation indicated that the dynamic trends in NLR and CT manifestation changes are highly valuable in predicting adverse outcomes of COVID-19 in the early stages of the disease, and this may also apply to other viral pneumonia. The prevalence of severe COVID-19 in our cohorts was approximately 31.15%, exceeding the rates reported in contemporaneous publications (10, 14). Conversely, in patients without enlarged pneumonia lesions (*n* = 32), the rate of severe illness was 6.25%, which was lower than reported. The difference was statistically significant (*p* = 0.006) in our cohort. This indicated that patients with increased lung lesions in the early days are more likely to progress severe illness than those with stable lung lesions. It is worth noting that there were no differences observed in changes in CPR and NLR between the two groups (Supplementary Table 1), suggesting that CT exhibits higher sensitivity than CPR and NLR in displaying the development of COVID-19.

In our study, the nomogram demonstrated excellent performance with an AUC of 0.961 and exhibited good calibration. Notably, a significant difference in predictive efficacy was presented between the mixed model incorporating categorical variables and the one incorporating continuous variables. This difference may arise due to variations in either the development patterns of lesions or the stage of COVID-19 pneumonia in patients.

In the early stage, GGO is the most common CT finding and usually develops rapidly (23, 24). Consolidation had also been observed, enlarging in some cases (1). In the field of pathology, GGO indicates interstitial thickening or alveolar damage, with airspaces being partially filled with inflammatory exudation (25). Consolidation, on the other hand, may be attributed to the presence of cellular fibromyxoid exudates in the alveoli (26). Disruption of the basement membrane and activation of fibroblasts increase the risk of patients progressing to ARDS. Pathologically, moderate to severe COVID-19 pneumonia is characterized by extensive mixed-to-consolidation-dominant lesions. These lesions are indicative of diffuse alveolar damage or acute fibrinous organizing pneumonia patterns, which are more prevalent in comparison to mild cases of COVID-19 (27). Logistic regression analysis revealed that the changing volume of SV and CV was an independent prognostic factor, while GV did not show a significant association with prognosis. These findings align with the observed pathological changes.

**Figure 4:**
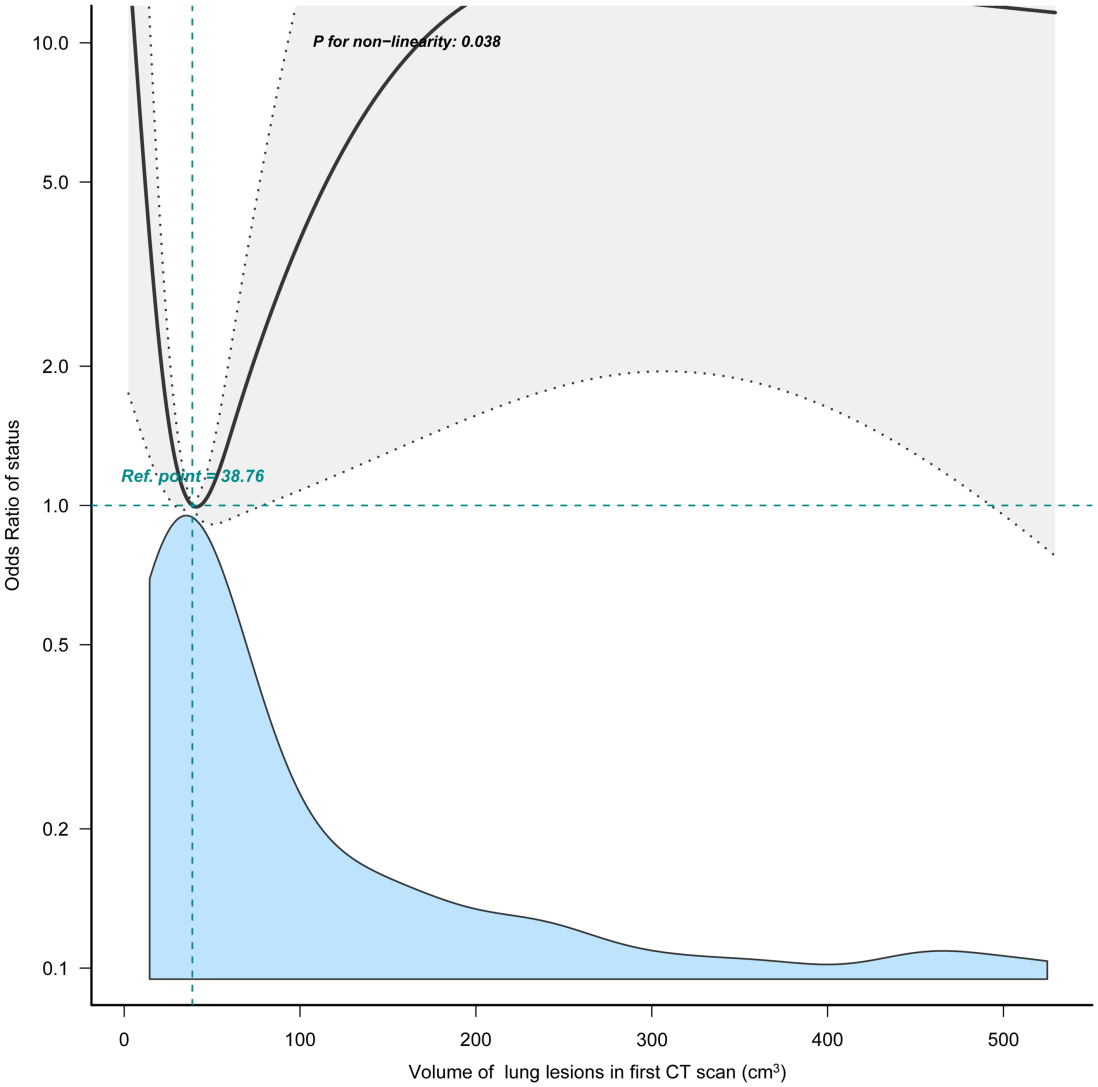
Relationship between the volume of lung lesions of first CT scan and progression to severe illness.

In our study, the volume of lung lesions between the two groups had no statistical difference (*p* = 0.907), and the relationship between the volume of lung lesions in the initial CT scan and the severity of the illness was non-linearity (*p* = 0.038) (Figure 4). This indicated that development patterns, but not the initial volume of lung lesions, affected the progress of the disease most.

To assess the models beyond mathematical performance metrics such as AUC, we used DCA to evaluate the predicted net benefit across various risk thresholds and examine the effect of different thresholds (28). DCA demonstrated that within the range of threshold probabilities from 0.01 to 0.81, the implementation of the nomogram in our study for predicting COVID-19 pneumonia yielded greater benefits compared to the “treat all” or “treat none” strategies.

Our study had a few limitations. First, due to the retrospective study design, the evaluation of patients who underwent serial CT scans was conducted. These scans were not based on a predefined protocol but rather ordered according to clinical necessity, which introduces a potential selection bias. Second, this study was conducted at a single center with a small sample size. The challenges posed by the ongoing mutation of the virus and the global pandemic have made it difficult to organize prospective multicenter trials specifically targeting patients infected with the original strain of SARS-CoV-2. To ensure model stability, we conducted internal verification through 200 iterations of bootstrap resampling. Third, there are some missing data in our study. Due to the insufficient availability of inspections, not all CRP data could be collected. Additionally, one patient’s NLR data were missing as they had their examination conducted in another hospital, which was not recorded in the HIS. However, we mitigated this issue (CRP) by using multiple imputations and replacing the missing value (NLR) with the median value. Moreover, CRP was not included in the nomogram analysis. Finally, all CT images analyzed in this study were obtained from the same CT scanner (Siemens SOMATOM Emotion, Germany). To validate and confirm the findings of this research, further investigations utilizing data from multiple sites and diverse scanners are warranted.

## 5 Conclusion

In summary, we have developed a quantitative prediction model that can assess the risk of patients. This model has the potential to guide the systematic treatment approach for admitted COVID-19 pneumonia patients in the early stages and aid in reducing the strain on healthcare resources and improving patient outcomes. COVID-19 pneumonia has many similarities with other types of viral infection and organized pneumonia in imaging and pathology (27). This result may serve as a reference to other viral infections in future.

## Data availability statement

The raw data supporting the conclusions of this article will be made available by the authors, without undue reservation.

## Ethics statement

The studies involving humans were approved by Ethics Committee of Jingzhou Central Hospital, Hubei, China. The studies were conducted in accordance with the local legislation and institutional requirements. The ethics committee/institutional review board waived the requirement of written informed consent for participation from the participants or the participants’ legal guardians/next of kin because the study is retrospective and is part of a public health outbreak investigation.

## Author contributions

LC: Writing – original draft, Software, Investigation, Funding acquisition, Data curation. ML: Writing – review & editing, Investigation, Funding acquisition. ZW: Writing – review & editing, Methodology, Investigation. SL: Writing – review & editing, Resources, Project administration. YH: Writing – review & editing, Supervision, Project administration, Funding acquisition.

## References

[1] Li X Zeng W Li X Chen H Shi L Li X. CT imaging changes of corona virus disease 2019(COVID-19): a multi-center study in Southwest China. J Transl Med. (2020) 18:154. doi: 10.1186/s12967-020-02324-w, PMID: 32252784 PMC7132551

[2] Yuan Y Jiao B Qu L Yang D Liu R. The development of COVID-19 treatment. Front Immunol. (2023) 14:1125246. doi: 10.3389/fimmu.2023.1125246, PMID: 36776881 PMC9909293

[3] Li M Lei P Zeng B Li Z Yu P Fan B. Coronavirus disease (COVID-19): Spectrum of CT findings and temporal progression of the disease. Acad Radiol. (2020) 27:603–8. doi: 10.1016/j.acra.2020.03.003, PMID: 32204987 PMC7156150

[4] Pan Y Guan H Zhou S Wang Y Li Q Zhu T. Initial CT findings and temporal changes in patients with the novel coronavirus pneumonia (2019-nCoV): a study of 63 patients in Wuhan, China. Eur Radiol. (2020) 30:3306–9. doi: 10.1007/s00330-020-06731-x, PMID: 32055945 PMC7087663

[5] Cereser L Da Re J Zuiani C Girometti R. Chest high-resolution computed tomography is associated to short-time progression to severe disease in patients with COVID-19 pneumonia. Clin Imaging. (2021) 70:61–6. doi: 10.1016/j.clinimag.2020.10.037, PMID: 33125986 PMC7585631

[6] Chassagnon G Vakalopoulou M Battistella E Christodoulidis S Hoang-Thi T-N Dangeard S. AI-driven quantification, staging and outcome prediction of COVID-19 pneumonia. Med Image Anal. (2021) 67:101860. doi: 10.1016/j.media.2020.101860, PMID: 33171345 PMC7558247

[7] Wang R Jiao Z Yang L Choi JW Xiong Z Halsey K. Artificial intelligence for prediction of COVID-19 progression using CT imaging and clinical data. Eur Radiol. (2022) 32:205–12. doi: 10.1007/s00330-021-08049-8, PMID: 34223954 PMC8256200

[8] Cheng Z Qin L Cao Q Dai J Pan A Yang W. Quantitative computed tomography of the coronavirus disease 2019 (COVID-19) pneumonia. Radiol Infect Dis. (2020) 7:55–61. doi: 10.1016/j.jrid.2020.04.004, PMID: 32346594 PMC7186132

[9] Gong J Ou J Qiu X Jie Y Chen Y Yuan L. A tool for early prediction of severe coronavirus disease 2019 (COVID-19): a multicenter study using the risk nomogram in Wuhan and Guangdong, China. Clin Infect Dis. (2020) 71:833–40. doi: 10.1093/cid/ciaa443, PMID: 32296824 PMC7184338

[10] Liu F Zhang Q Huang C Shi C Wang L Shi N. (2020). CT quantification of pneumonia lesions in early days predicts progression to severe illness in a cohort of COVID-19 patients. Available at: https://www.thno.org/v10p5613.pdf (Accessed April 15, 2023). 10.7150/thno.45985 PMC7196293 32373235

[11] Wu Q Wang S Li L Wu Q Qian W Hu Y. Radiomics analysis of computed tomography helps predict poor prognostic outcome in COVID-19. Theranostics. (2020) 10:7231–44. doi: 10.7150/thno.46428, PMID: 32641989 PMC7330838

[12] Fang X Li X Bian Y Ji X Lu J. Radiomics nomogram for the prediction of 2019 novel coronavirus pneumonia caused by SARS-CoV-2. Eur Radiol. (2020) 30:6888–901. doi: 10.1007/s00330-020-07032-z, PMID: 32621237 PMC7332742

[13] Liu F Zhang Q Huang C Shi C Wang L Shi N. CT quantification of pneumonia lesions in early days predicts progression to severe illness in a cohort of COVID-19 patients. Theranostics. (2020) 10:5613–22. doi: 10.7150/thno.45985, PMID: 32373235 PMC7196293

[14] Feng Z Yu Q Yao S Luo L Zhou W Mao X. Early prediction of disease progression in COVID-19 pneumonia patients with chest CT and clinical characteristics. Nat Commun. (2020) 11:4968. doi: 10.1038/s41467-020-18786-x, PMID: 33009413 PMC7532528

[15] China NHC (2024). Diagnosis and treatment protocols of pneumonia caused by novel coronavirus (trial version 9). Available at: https://www.gov.cn/zhengce/zhengceku/2022-03/15/content_5679257.htm (Accessed January 26, 2024).

[16] Metlay JP Waterer GW Long AC Anzueto A Brozek J Crothers K. Diagnosis and treatment of adults with community-acquired pneumonia. An official clinical practice guideline of the American Thoracic Society and Infectious Diseases Society of America. Am J Respir Crit Care Med. (2019) 200:e45–67. doi: 10.1164/rccm.201908-1581ST, PMID: 31573350 PMC6812437

[17] Wang S Zhou M Liu Z Liu Z Gu D Zang Y. Central focused convolutional neural networks: developing a data-driven model for lung nodule segmentation. Med Image Anal. (2017) 40:172–83. doi: 10.1016/j.media.2017.06.014, PMID: 28688283 PMC5661888

[18] Ronneberger O Fischer P Brox T. U-net: convolutional networks for biomedical image segmentation In: Navab N Hornegger J Wells WM Frangi AF, editors. Medical Image Computing and Computer-Assisted Intervention—MICCAI 2015. Lecture Notes in Computer Science. Cham: Springer International Publishing (2015). 234–41.

[19] Suzuki K Kusumoto M Watanabe S Tsuchiya R Asamura H. Radiologic classification of small adenocarcinoma of the lung: radiologic-pathologic correlation and its prognostic impact. Ann Thorac Surg. (2006) 81:413–9. doi: 10.1016/j.athoracsur.2005.07.058, PMID: 16427823

[20] Wang D Hu B Hu C Zhu F Liu X Zhang J. Clinical characteristics of 138 hospitalized patients with 2019 novel coronavirus-infected pneumonia in Wuhan, China. JAMA. (2020) 323:1061–9. doi: 10.1001/jama.2020.1585, PMID: 32031570 PMC7042881

[21] Abkhoo A Shaker E Mehrabinejad M-M Azadbakht J Sadighi N Salahshour F. Factors predicting outcome in intensive care unit-admitted COVID-19 patients: using clinical, laboratory, and radiologic characteristics. Crit Care Res Pract. (2021) 2021:9941570–7. doi: 10.1155/2021/9941570, PMID: 34306751 PMC8285200

[22] Jimeno S Ventura PS Castellano JM García-Adasme SI Miranda M Touza P. Prognostic implications of neutrophil-lymphocyte ratio in COVID-19. Eur J Clin Investig. (2021) 51:e13404. doi: 10.1111/eci.13404, PMID: 32918295

[23] Simpson S Kay FU Abbara S Bhalla S Chung JH Chung M. Radiological society of North America expert consensus document on reporting chest CT findings related to COVID-19: endorsed by the Society of Thoracic Radiology, the American College of Radiology, and RSNA. Radiol Cardiothorac Imaging. (2020) 2:e200152. doi: 10.1148/ryct.2020200152, PMID: 33778571 PMC7233447

[24] Pan F Ye T Sun P Gui S Liang B Li L. Time course of lung changes at chest CT during recovery from coronavirus disease 2019 (COVID-19). Radiology. (2020) 295:715–21. doi: 10.1148/radiol.2020200370, PMID: 32053470 PMC7233367

[25] Zhou S Chen C Hu Y Lv W Ai T Xia L. Chest CT imaging features and severity scores as biomarkers for prognostic prediction in patients with COVID-19. Ann Transl Med. (2020) 8:1449. doi: 10.21037/atm-20-3421, PMID: 33313194 PMC7723645

[26] Delorey TM Ziegler CGK Heimberg G Normand R Yang Y Segerstolpe Å. COVID-19 tissue atlases reveal SARS-CoV-2 pathology and cellular targets. Nature. (2021) 595:107–13. doi: 10.1038/s41586-021-03570-8, PMID: 33915569 PMC8919505

[27] Lee JH Koh J Jeon YK Goo JM Yoon SH. An integrated radiologic-pathologic understanding of COVID-19 pneumonia. Radiology. (2023) 306:e222600. doi: 10.1148/radiol.222600, PMID: 36648343 PMC9868683

[28] Vickers AJ Elkin EB. Decision curve analysis: a novel method for evaluating prediction models. Med Decis Mak. (2006) 26:565–74. doi: 10.1177/0272989X06295361, PMID: 17099194 PMC2577036

