# Supplementary material for: A nomogram to predict severe COVID-19 patients with increased pulmonary lesions in early days
Source: Front Med (Lausanne). 2024 Apr 26;11:1343661. doi: 10.3389/fmed.2024.1343661 (PMC11082326; doi:10.3389/fmed.2024.1343661)
Supplement: Supplementary file 1 [file Table_1.docx]

| Supplement table 1 Clinical and CT quantitative characteristics of COVID-19 patients * | | | | |
| --- | --- | --- | --- | --- |
| Variables | Total  (n = 93) | Negative CT change  (n = 32) | Positive CT change  (n = 61) | p |
| Progression, n (%) |  |  |  | 0.006 |
| no | 72 (77.4) | 30 (93.8) | 42 (68.9) |  |
| yes | 21 (22.6) | 2 (6.2) | 19 (31.1) |  |
| Age,year,(IQR) | 43.0 (31.0, 55.0) | 39.0 (30.0, 52.8) | 44.0 (34.0, 55.0) | 0.352 |
| Gender, n (%) |  |  |  | 0.598 |
| male | 50 (53.8) | 16 (50) | 34 (55.7) |  |
| female | 43 (46.2) | 16 (50) | 27 (44.3) |  |
| Comorbidity, n (%) |  |  |  | 0.43 |
| no | 68 (73.1) | 25 (78.1) | 43 (70.5) |  |
| yes | 25 (26.9) | 7 (21.9) | 18 (29.5) |  |
| Interval, day, (IQR) | 3.0 (3.0, 4.0) | 4.0 (3.0, 4.0) | 3.0 (3.0, 4.0) | 0.721 |
| **Changes in 7 days (IQR)** | |  |  |  |
| GV (cm^3^) | 16.2 (-0.8, 42.7) | -3.2 (-20.3, 1.6) | 36.4 (15.1, 54.4) | < 0.001 |
| SV (cm^3^) | 11.8 (0.5, 44.9) | -4.0 (-20.3, 2.0) | 25.4 (10.8, 63.2) | < 0.001 |
| CV (cm^3^) | 3.7 (-1.0, 25.9) | -1.1 (-8.7, 0.3) | 12.2 (2.0, 41.9) | < 0.001 |
| NLR | 1.6 (-0.5, 8.1) | 1.1 (-0.8, 6.2) | 2.1 (-0.4, 12.3) | 0.222 |
| CRP | -2.3 (-12.1, 1.5) | -2.2 (-11.4, 0.0) | -2.5 (-12.1, 7.7) | 0.198 |
| *the cohort was divided based on whether the volume of lung lesions increased by more than 10cm^3^. | | | | |
