# Supplementary figures and images for: A nomogram to predict severe COVID-19 patients with increased pulmonary lesions in early days
Source: Front Med (Lausanne). 2024 Apr 26;11:1343661. doi: 10.3389/fmed.2024.1343661 (PMC11082326; doi:10.3389/fmed.2024.1343661)

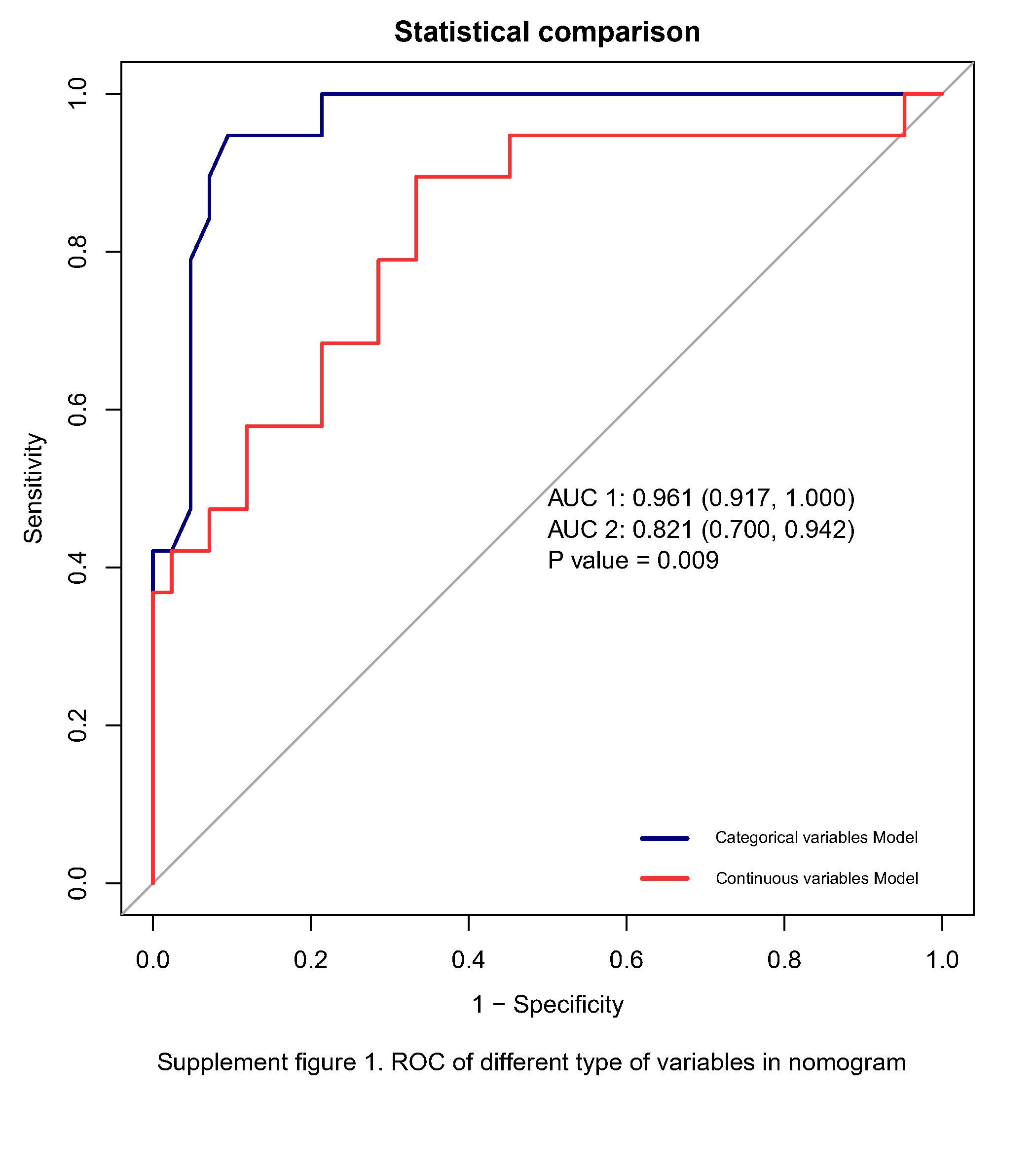

Supplement: Supplementary file 2 [file Image_1.TIFF]
